# Bio-Inspired Highest Lift-to-Drag-Ratio Fin Shape and Angle for Maximum Surfboard Stability: Flow Around Fish Fins

**DOI:** 10.3390/biomimetics10040234

**Published:** 2025-04-09

**Authors:** Megan S. MacNeill, Brian D. Barkdoll

**Affiliations:** Civil, Environmental, and Geospatial Engineering Department, Michigan Technological University, Houghton, MI 49931, USA

**Keywords:** surfboard, drag force, lift force, optimization, fish

## Abstract

Wave surfing is a multi-billion dollar industry involving both maneuverability and speed, yet little research has been performed regarding the highest lift-to-drag-ratio fin shape for these competing qualities. Numerical modeling and laboratory experiments were performed here to identify a bio-inspired fin shape that maximized lateral stability and minimized drag forces, in order to increase surfing maneuverability. Nine fins based on dorsal fins of real fish were tested. Both the CFD and laboratory experiments confirmed that the fin of the same shape as that of the Short-Finned Pilot Whale at an angle of attack of 10° had the greatest lift-to-drag ratios. Flow patterns around fins at a low angle of attack were smooth with negligible flow separation, while at any angle of attack greater than 25°, flow-separation-induced drag forces became excessive.

## 1. Introduction

Background. As a popular sport worldwide, surfing is a global industry that generates billions of dollars annually. Although surfing has been practiced for many years, it was not until the 1930s that the first fin was introduced. Many fin sizes, shapes, materials, and setups are on the market for specific wave types, as well as riders of different weights, surfing styles, and preferences.

Traditionally, surfboard designers and professional surfers work together to improve the designs of surfboards and fins, but there are problems with this method. It is common for several variables to simultaneously change during the shape-testing process, thereby making the highest lift-to-drag-ratio shape and angle difficult to ascertain. Each surfer also has a different preference and style, which leads to inconsistencies in the design feedback. Speed, maneuverability, and control are major considerations in design during the shaping process [1,2]. The pertinent forces are lift and drag, which are related to the fluid properties, object shape, size, and wetted perimeter roughness characteristics. Lift refers to fluid forces acting transverse to the fluid flow direction, while drag forces act along the flow direction. The profile shape of a fin might have a dramatic effect on the performance [3]. Many surfboard fin shapes have come from the fin features of aquatic mammals and fish [4]. The emulation of biological characteristics can improve the design and performance of a surfboard fin [5]. There have only been a few scientific studies on surfboard fin design using computational or experimental analysis of lift and drag force results.

**PURPOSE OF THE FIN.** Surfboard fins are similar to a sailboat skeg and keel. The main purposes of these attributes are to provide directional stability and control of the watercraft. The keel of a sailboat is a hydrodynamic element. It is used to counteract the sideways force of wind and convert it into a forward motion. As the boat moves forward, the keel generates lift [6]. Surfboard fins counteract the sideways force of the surfer against the forward velocity of the wave. The fins also provide lift to the surfboard and rider, thereby making sharp turns and maneuvering up and down the wave possible.

The dorsal fin (s) is located on the backs of many marine mammals and fish. The rudimentary function of a fish or aquatic mammal’s dorsal fin is to provide directional stability [7]. It keeps the swimming species from rolling while swimming [8]. The secondary purpose is to allow quick stopping and turning. The shape of this fin can also help generate thrust [7]. A dorsal fin shape and body location is an individual characteristic dependent on species, sex, and the individual [8]. Another important hydrodynamic function is the angle of attack of the dorsal fin, which is a strong variable in determining vortex characteristics [8].

**Figure 1:**
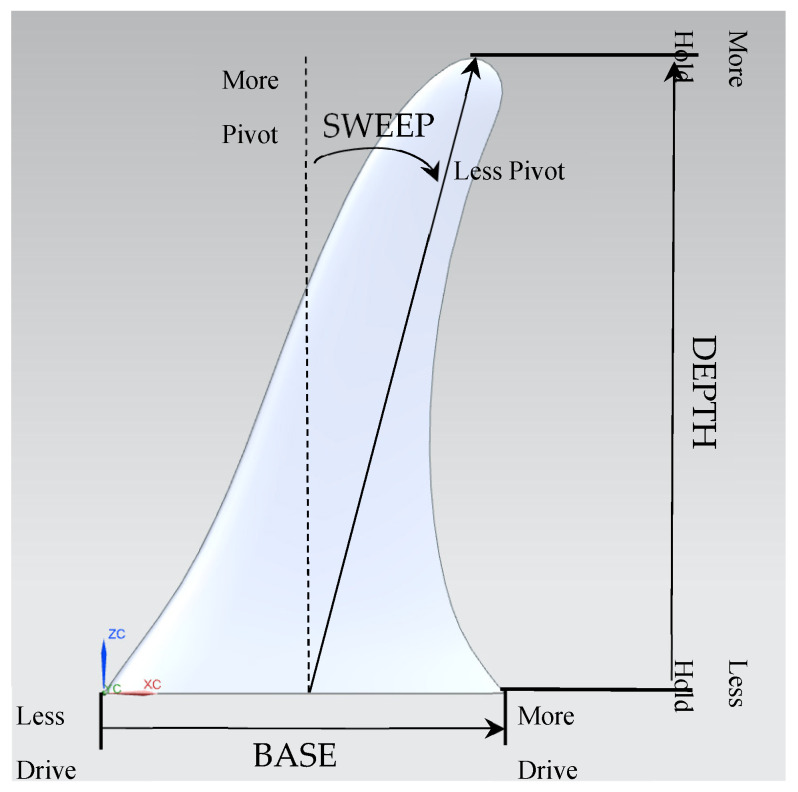
Basic design features of a surfboard fin.

The shape, material, and size of a fin are important aspects in the design process. By altering one variable, the hydrodynamic effects will change and reflect in the surfer’s performance. The base of a fin is the area in which the fin connects to the surfboard (Figure 1). Its length is measured from the leading edge to the trailing edge of the foil shape. It is linked to the surfer’s ability to accelerate. This means that the greater the base length, the faster acceleration the fin can provide to the surfboard. Fin depth is how deeply the fin penetrates the water. Its length is measured from the base to the tip of the fin. It is believed that a longer fin depth provides more hold, which allows the surfer to have more lateral stability. As fin heights are reduced, it could be easier for undesirable lateral board sliding to occur. Fins have similarities to airplane wings. The aspect ratio, for example, influences the lift and drag components. Aspect ratio is the proportional relationship of the wing’s span length to chord measurement [9]. High-aspect-ratio wings have a longer span (fin depth) and a smaller chord length (fin base). Generally, this provides a higher performance wing with less drag compared to a low-aspect-ratio wing [10]. The three-dimensional fin is made up of two-dimensional hydrofoil shapes across the span of the fin. The fin’s shape commonly has a moderate to high aspect ratio.

Sweep, also known as rake, is thought to influence the pivoting motion. It is the angle from the mid-base point to the tip of the fin. A fin with a lower-angled rake means the surfer can make tighter turns. Consequently, this might deliver a fin that is less aerodynamic because the leading edge is blunter to the oncoming water.

The total surface area of a fin can improve a surfer’s stability and hold during turns. It does, however, provide more drag due to a high wetted area. For this reason, the surface of the fin must be smooth in order to reduce surface tension and ultimately drag forces. The modern fin can be made from a combination of advanced materials and constructed in various arrangements. Materials can include glass epoxy, fiberglass, carbon fiber, wood, Texalium, etc. The material design can also provide different levels of distortion. This flex in the fin is dependent on the material type and structure. It is the ability of the material to bend, caused by lateral pressure when turning. The stiffer the fin, the more instant response a rider can have to provide speed and drive down a wave.

Surfboards can have multiple fins. The outside fins, or rail fins, can be tilted towards the outside of the board at some ‘cant’ angle. The higher the cant angle, the greater the maneuverability a rider will have. Less cant is thought to provide faster acceleration. The toe angle (angle of attack) is the angle that the fin has with the surfboard longitudinal centerline (stringer). The center fin is always aligned with the stringer (angle of attack of zero).

***LIFT AND DRAG.*** Lift occurs when an object in a moving fluid changes the flow direction [11,12,13]. As a result, the object will lift in the opposite direction. Both lift and drag are dependent on the Reynolds number [14]. Lift is a hydrodynamic force, described by a vector quantity, generated by the motion of the fin through water. Airfoil theory states that the direction of lift is through the center of pressure of the fin, perpendicular to the flow direction. Magnitude is dependent on the geometry of the fin setup, the angle of attack, and the fluid motion and characteristics. This lifting action provides a side force necessary to hold the surfboard for directional stability and enhance maneuverability [11,15].

The fin on a surfboard is a foiled shape, which provides the surfer lateral lift to maneuver and change the direction of the board [16]. The airstream shape of the fin induces two physical responses to create lift: a positive lifting pressure from below the hydrofoil and a negative lifting pressure from above the hydrofoil [10]. Shear stress is due to the frictional effects of a fluid passing over the surface of the hydrofoil and is measured tangentially on the surface of the hydrofoil [9].

For a symmetric hydrofoil at zero angle of attack, the speed and pressure changes are symmetric on both sides of the hydrofoil. As the angle of attack is increased, the flow lines experience constriction that causes an increase in flow velocity. At a positive angle of attack the hydrofoil produces lower pressure on the topside (suction side) compared to the bottom side (compression side). This is due to a constricted flow path on the suction side, mutually decreasing the static pressure and increasing the dynamic pressure [10]. These pressure and velocity differences allow for the fluid to circulate from low-pressure to high-pressure areas at its sharp trailing edges.

Amplifying the angle of attack improves the lifting force. For all hydrofoils, there is a critical inclination angle. When exceeded, turbulence will induce drag and the lifting forces will drop [16].

***LIFT AND DRAG COEFFICIENTS.*** Lift and drag coefficients encompass the hydrodynamic components between the water and fin interaction [11]. This includes water density, ρ, object velocity (free-stream velocity), *U*_∞_, object frontal area, *A*, and lift or drag force values, *F_L_* and *F_D_*. Total drag can be described by calculating the drag coefficient *C_D_*, while the lift coefficient *C_L_* is used to express the total lift of the fin [14]. Both the lift and drag coefficients are non-dimensional values, given as [15]

*C_L_ = F_L_ρU_∞_*^2^*A*/2   *C_D_ = F_D_ρU_∞_*^2^*A*/2 (1)

The frontal area is important when calculating lift and drag coefficients [11]. The front of the fin shows the leading edge, and the back of the fin shows the trailing edge. The chord length (fin base) is the distance from the leading to trailing edge (Figure 1). This length changes throughout the span (fin depth) of the fin.

The lift-to-drag ratio (L/D) is also another tool used to determine a desirable fin shape or orientation to allow for both speed and maneuverability. A higher L/D ratio indicates a more efficient fin, meaning the fin with the greatest amount of lift and least amount of drag [13].

**LITERATURE REVIEW.** Carswell and Lavery [3] published one of only a few known archival studies on the topic of surfboard fin design and performance. CAD tools and CFD modeling were used in the development and testing of three symmetric single fins. Each fin had the same side and front profiles, but the spanwise direction differed with three base foils investigated: a standard foil, a NACA 4-series, and a NACA 6-series. The fins were tested seperately using the commercially available computational fluid dynamics (CFD) program FLUENT [13] by setting the inlet velocity to 0.6–0.7 m/s and varying the angles of attack from 0° to 12°. Turbulence intensity was set to 1% in the k-epsilon turbulent flow model. Lift, drag, and pressure coefficients were calculated and compared. The geometry of the fins and flow parameters also differed. It was concluded that the 4-series and 6-series fins produced higher lift and lower drag values compared to the standard foil shapes. It was suggested that the profile design might provide a greater impact on the performance of the fin over foil design.

Carswell [17] performed a comprehensive study on the hydrodynamics of surfboard fins in which both CFD and laboratory experiments were utilized to find lift and drag properties of commercially available flexible surfboard fins. This fluid–structure interaction study revealed that allowing the rear of the fin to flex improves the lift and drag properties of the fin. It was also found that a high-aspect-ratio fin exhibited a faster decrease in lift with increasing drag subsequent to stall conditions. Bio-inspired fin shapes were not studied directly, but the fins studied did somewhat resemble fish dorsal fin shapes. The CFD tool was described also in Carswell et al. [17].

Lavery et al. [17] wrote another report based on the effects of filets on fins. The filets investigated were an un-fileted fin and filets with radii of 5 mm, 10 mm, 15 mm, and 20 mm. Three setups were examined at angles of attack ranging from 0° to 25°: a single symmetric center fin, a single side fin, and a thruster setup. Inlet velocities ranged from 1 mps to 7 mps. A CAD program called Dat98 and CFD modeling were used for acquiring the lift, drag, and pressure data. It was found that the addition of filets provided little improvement to the overall drag of the fins.

A CFD study was performed through the investigation of three- and four-fin setup configurations by Gudimetla et al. [12]. The ANSYS CFX Siemen’s NX 9.0 [18] flow solver was used to measure the lift and drag forces, as well as flow visualization around the fins. These results were compared to the seven-equation Reynolds stress (RSM) turbulence model. The three-fin setup produced higher lift and coefficients at lower angles of attack, while the four-fin setup had lower lift and drag values at smaller angles of attack. It was concluded that the three-fin design was more efficient, as evidenced by higher L/D ratios, which would be most applicable for surfing typical waves. The four-fin design was more suitable for big-wave surfing, because the low lift and drag values made the configuration more stable. This was possibly due to the existence of tip vortices.

Sakamoto and Yanamoto [4] compared a conventional fin design to three different modified fins. L/D ratios were used to evaluate the thruster configurations by modeling them in a CFD program called Engineering Fluid Dynamics Proengineer. The computational results were compared to visual flow experiments completed in a water tank and by surfing the fin in the ocean. The quantitative L/D ratios did not provide usable results, however.

**RESEARCH GAP.** No study has studied bio-inspired fin shapes for surfboards, like the present one.

**PROBLEM STATEMENT.** The problem to be solved is to find the highest lift-to-drag-ratio single-fin shape and angle of attack to maximize maneuverability and speed.

**USEFULNESS OF THE STUDY.** Knowing the optimal bioinspired surfboard and the optimal angle of attack to maximize the lift-to-drag ratio helps surfers obtain speed and maneuverability.

**Figure 2:**
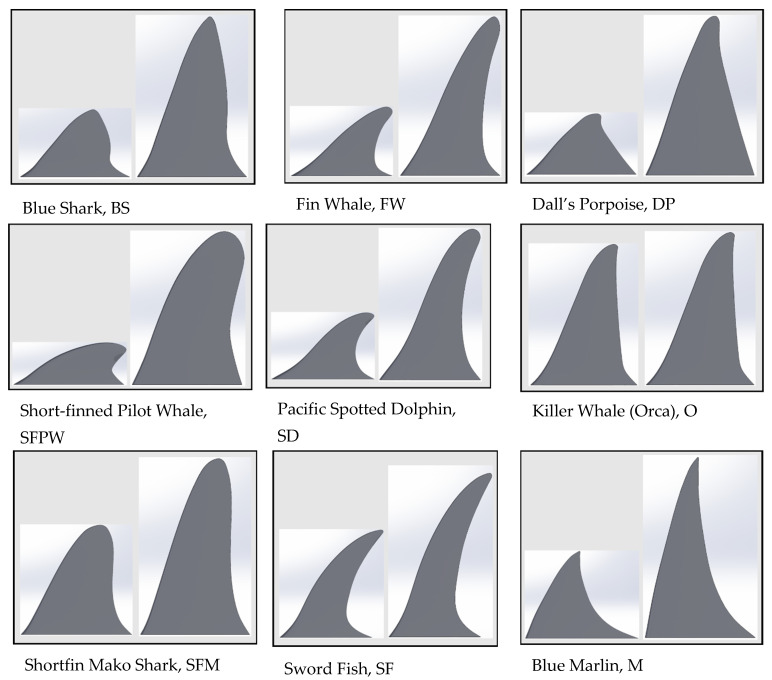
Fin shapes tested (original shape on left; homogenized aspect ratio on right).

**BIO-INSPIRED DORSAL FIN PROFILES.** The nine fins chosen for this project were based on species that are considered some of the fastest swimming aquatic mammals and fish in the world [19]. A variety of fish, sharks, dolphins, and whales were used to compare vastly different dorsal fin shapes (Figure 2).

## 2. Procedure

Various bio-inspired fin shapes were studied both numerically and experimentally at various angles of attack with zero cant angles and no deflection, for simplicity. The numerical and experimental results were not identical and, therefore, were not meant to be compared but instead to study the problem in two different ways. Since the purpose of the present study was to find the fin with the highest lift-to-drag ratio and not to validate experimental approaches or a CFD model, it was decided that two independent methods (CFD and experiments) would be used to find the best fin shape. Fin aspect ratios were homogenized (Figure 2) to allow for direct comparability of fin shape, the variable being considered here, and not aspect ratio. The importance of air concentration, void fraction, and cavitation numbers as real surfboard conditions were beyond the scope of this work but should be considered for future study.

**Figure 3:**
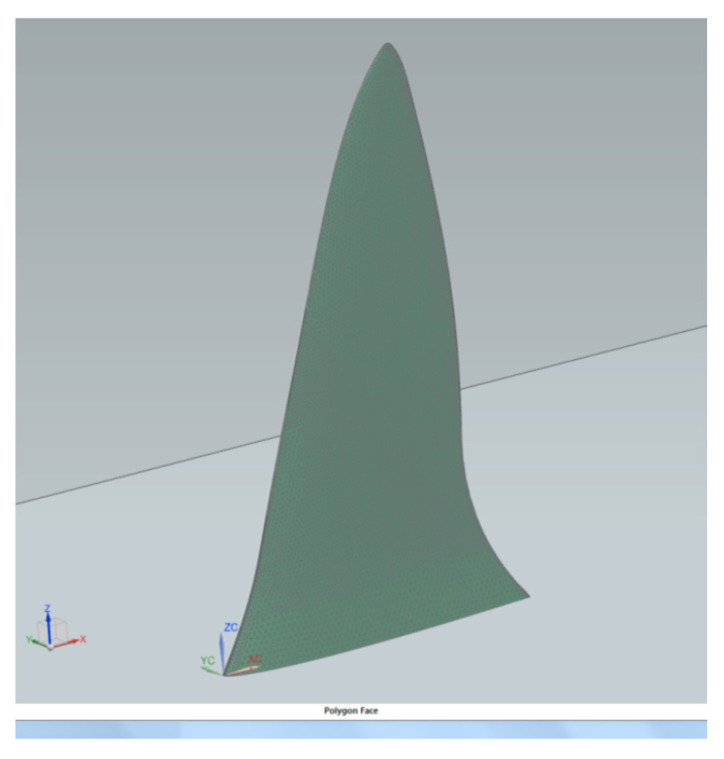
Meshing of the Blue Shark fin.

***CFD Modeling.*** All fin design modeling and simulations were performed on a Dell Precision T1700 computer with a Windows 7 Enterprise 64-bit operating system. The system operated on an Intel^®^ Core™ i7-4770 CPU processor at 3.40 GHz and 8.00 GB of RAM. SOLIDWORKS Siemen’s NX 9.0 three-dimensional solid modeling computer-aided design (CAD) software [20] was used in the design of the 3D fins. Each fin was equipped with a series of two-dimensional hydrofoils attached to a spline. The “Loft” tool was used in order to project these 2D drawings into a 3D model. Siemen’s NX 9.0 [20] was used in order to compute the fluid flow, the flow field, as well as lift and drag forces for angles of attack ranging from 0° to 45°. The well-known time-averaged Reynolds-averaged Navier–Stokes (RANS) k-epsilon model was applied to compute fluid velocities. The equations are given in [21]. This two-equation model is a type of linear eddy viscosity (LEV) model that solves turbulent flow fields. Grid dependency studies were conducted for each CFD run that gradually refined the tetrahedral computational mesh (TET10) until finer meshes did not result in changes in the lift and drag forces more than 0.05%. A final fin mesh of 2 mm was chosen, resulting in a range of grid points from 3.9 × 10^4^ to 8.0 × 10^4^. A fluid domain mesh of a relative element size of 0.0045 or 5.4 mm was employed. Uniform flow upstream and downstream boundary conditions were used. Water values were employed to replicate typical values as follows: ρ = 997 kg/m^3^, T = 20 °C, μ = 1.002 × 10^−3^ kg/m/s, and υ = 1.004 × 10^−6^ m^2^/s. The fin wall roughness height was determined to be 1.5 microns to match that of typical surfboard fins. Wall functions were used to calculate the flow in the boundary layer with the fin surface to reduce the computational burden of resolving the boundary layer. The free-slip boundary conditions were used on the outside boundaries, while the no-slip boundary condition was used at the fin boundaries. To mesh each solid fin and fluid domain, the 3D tetrahedral meshing option was applied. Figure 3 shows the mesh for the Blue Shark fin, as an example.

**Figure 4:**
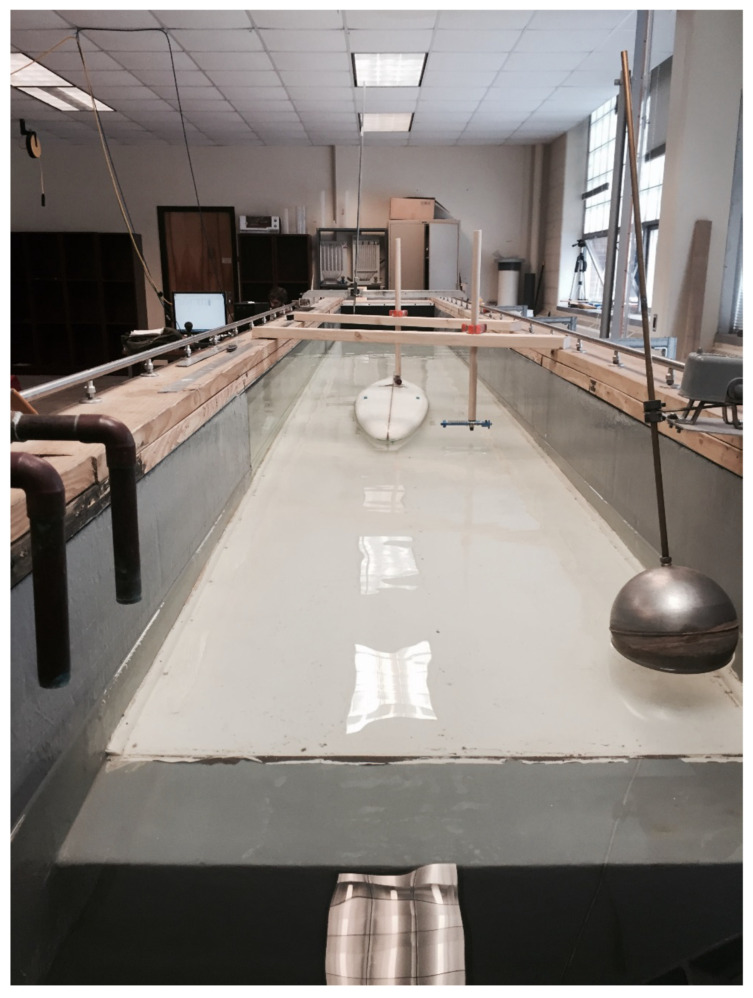
Experimental setup looking upstream.

***Experimental setup.*** Physical experiments were performed in a water channel consisting of a water inlet, flow straighteners, a flow-development section, testing zone, float valve depth controller, and an outlet zone (Figure 4). The inner-wall dimensions had a width of 0.92 m, a height of 1.11 m, and an inside length of 10.18 m. The return pipe was equipped with a manometer to measure the flow rate. A series of small-diameter flow-straightening pipes ensured fully developed flow by the test section. A rubber mat was installed 0.43 m from the flow straighteners to calm any water surface fluctuations. A flat bed was constructed following the flow straightener section, extending to the outlet section. The experimental section had a length of 8.04 m and stood 0.70 m in height at 0° longitudinal slope. The Cartesian coordinate system was defined as the positive X-direction downstream, positive Y direction transvers to flow, and the Z direction vertical.

The various fin shapes and angles of attack tested were attached to a small-scale surfboard based on that of a well-known board called the Gerry Lopez 1975 Lightning Bolt. This sleek longboard, intended for a single fin setup [22], had a length of 2.44 m and a width of 47.0 cm at the broadest point. The silhouette formed a medium rocker (bottom curvature) and a flat deck (top surface curvature). The nose was designed as a pin shape with a corresponding width dimension of 30.5 cm. The edges had soft rails (curved edges) across most of the surfboard’s length but developed into hard rails (less rounded edge) near the tail. The surfboard had a maximum thickness of 7.3 cm, tapering near the edges. The tail was molded into a flyer pintail shape at 22.9 cm wide. The initial single-fin position was placed 14.0 cm from the tail [23]. The surfboard was scaled down to fit the water channel. A 1:3 ratio of the surfboard-to-channel width was used. Using this ratio based on the water channel width (0.92 m), the surfboard width was set to 30.7 cm. All other surfboard dimensions were based off this 1:3 ratio, namely a length of 1.59 m and a width of 30.7 cm. The fin parameters consisted of an initial base length of 112 mm and 177.8 mm height.

The 3D printer used to print each fin and the fin insert system was the Ultimaker 2. The associated software program, Cura Siemen’s NX 9.0, was used to import the STL files for formatting purposes. The filament used for each print was a 2.85 mm diameter PLA (polylactic acid) polymer. The blue PLA plastic came on a reel with approximately 90 m of material. The print temperature was set to 210 °C with all fans turned on, while the plate temperature was set to 70 °C. For higher-quality results, the standard print speed was reduced to 50 mm/s. To save on time and material, the interior of the fins were set for a 20% infill; this value gave enough stability for the purpose of these experiments. Each fin print took approximately four to five hours per fin. Polylactic acid (PLA) is a thermoplastic material that is biodegradable [24]. PLA has a melting point at 200 °C, but it does harden once cooled [25]. This material was chosen based on its high strength and ease of processing to construct the fins. It was 90 m long with a 2.85 mm diameter and was packaged on a reel for printing convenience [26]. A light blue color was chosen for contrast against the floor of the water channel. Once the fins were printed, two coats of Sikkens Colorbuild Plus coating were applied [27] to provide a smooth surface to the fin and to seal the fin for water resistance.

Drag and lift forces were measured using separate calibrated spring-loaded force gauges in each direction. A syringe with an attached copper tube was used to inject dye in front of the fin. An iPhone 6 was used for photographing and videotaping the dye injection experiments. The camera on the iPhone 6 captured 1.5-micron pixel pictures and 240 fps slow motion video [28,29]. The velocity of water was measured using two systems: a two-tube manometer and a Nortek Vectrino Acoustic Doppler Velocimeter (ADV) that measures three-dimensional instantaneous velocity values by sending out an acoustic signal that reflects back from a measurement volume [30].

## 3. Results and Discussion

### CFD Modeling

**Figure 5:**
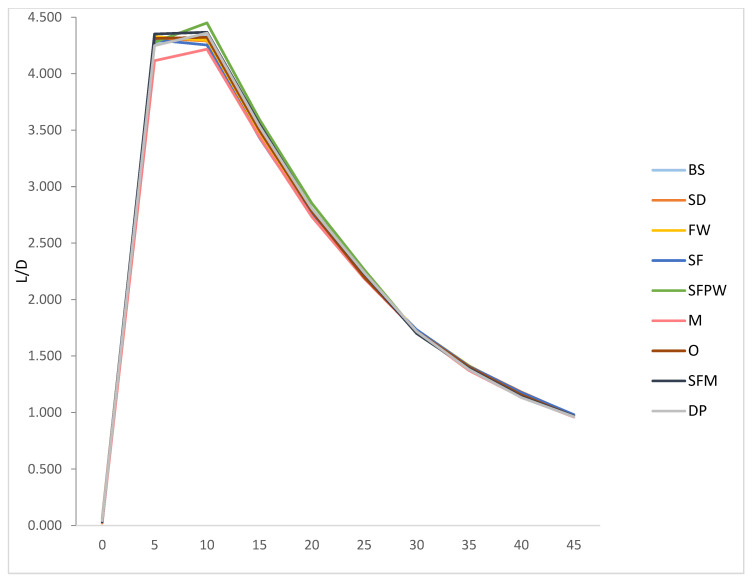
Lift-to-drag-ratio values from CFD data collected for nine fins at ten angles of attack (0–45°) showing that the SFPW has the highest ratio for angles from 10 to 25°.

The lift-to-drag ratio (L/D) provided a basis to decide which fin was the most efficient. Figure 5 provides calculated L/D ratios from collected CFD data for each of the nine fins (Figure 2). The shapes of the curves were similar to typical L/D ratio trends for symmetric hydrofoil 3D finite wings. L/D values varied greatly with angle of attack but not with fin shape. No single fin consistently showed high or low efficiency values for all angles of attack. The SD, FW, and SF each peaked at 5°, while the remaining fins peaked at 10°. Out of the ten angles of attack, the Marlin was the least efficient fin for six angles. The SFM shark was the most efficient fin at 5° but had the lowest L/D ratio at 30°. This also occurred for the FW and SF. The FW was the least efficient at 0° but most efficient at 30° and 35°. The SF had the lowest ratio at 15° but the highest at 45°. Notably, the SFPW had the highest L/D ratio for attack angles, from 10 to 25°. It had an L/D value of 4.449, while the next highest was SFM at 4.351.

**Figure 6:**
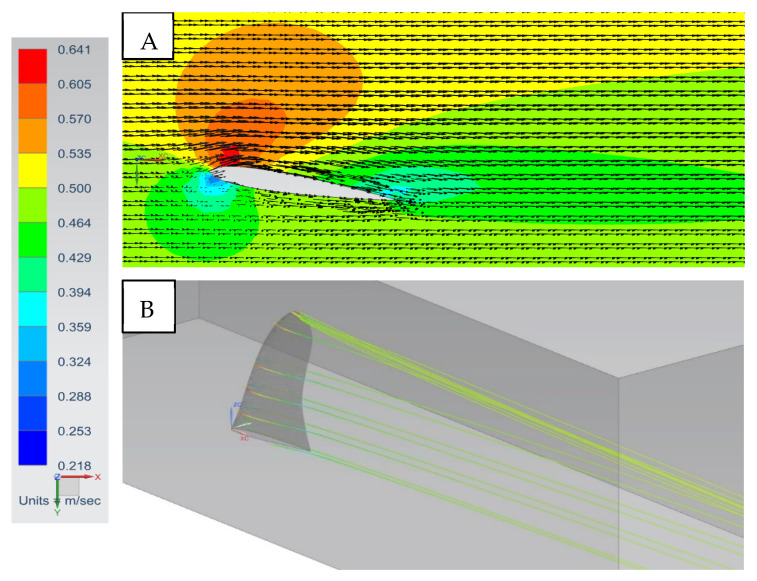
SFPW at α = 10°, *U*_∞_ = 0.5 mps. (**A**) Velocity magnitudes and flow vectors: plan view at the mid-section. (**B**) Streamlines: isometric view from trailing edge of the fin.

**Figure 7:**
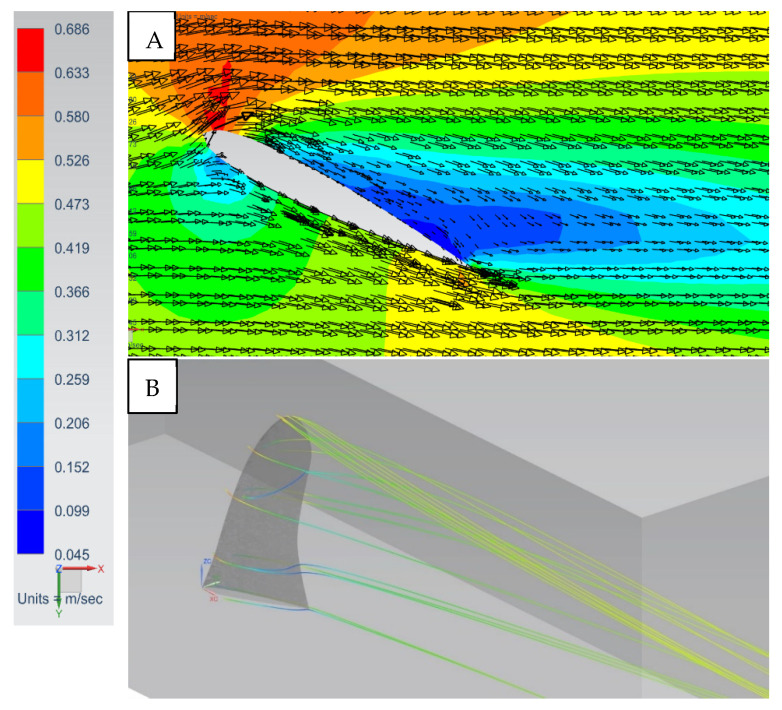
SFPW at α = 25°, *U*_∞_ = 0.5 mps. (**A**) Velocity magnitudes and flow vectors: plan view at the mid-section. (**B**) Streamlines: isometric view from trailing edge of the fin.

**Figure 8:**
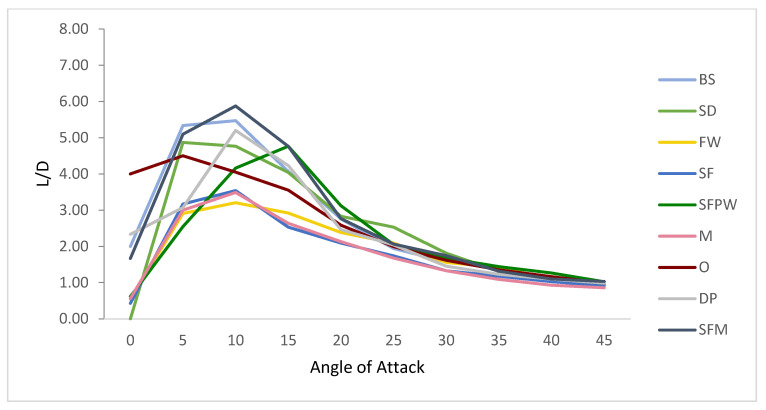
Lift-to-drag-ratio values from experimental data collected for nine fins at ten angles of attack (0–45°), showing that the SFPW has the highest ratio at angles of between 15, 20, 35, and 40°.

At a small angle of attack of 10° for the SFPW velocity, vectors and streamlines reveal slight flow separation and a tip vortex emanating from the fin (Figure 6). At an increased angle of attack of 25° there are exacerbated flow-separation-induced swirling eddies in the fin wake (Figure 7), thereby inducing high drag forces relative to the lift forces. In addition, the tip vortex strength was increased. This physically explains why the L/D ratio diminishes at angles of attack higher than 25° (Figure 8). Higher angles of attack resulted in increased flow separation and drag.

***EXPERIMENTAL MODELING.*** To achieve a complete understanding of the flow field around the SFPW, laboratory visual experiments were performed. These experiments were performed with fins differing from those in the numerical experiments mentioned above and, therefore, cannot be directly compared to the numerical results. Figure 8 provides calculated L/D ratios from measured experimental data for each of the nine fins. The shapes of the curves were similar to typical L/D ratio trends for symmetric hydrofoil 3D finite wings (fins). Starting from 0°, in sequential order, low efficiency values were generated from the SD, SFPW, FW, and the SF (15° and 20°). At higher angles of attack, the Marlin produced the lowest efficiency values. Excluding 35°, the Marlin was the least efficient between 25° and 45°. At 0°, the fin with the greatest ratio was the Orca, with a value of 4.00. The FW was the least efficient fin at 10°. At that same angle, the SFM shark produced the peak L/D ratio (5.88) for all nine fins and all ten angles of attack. The SFPW yielded the most peak L/D ratios at angles of 15° through 20° and 35° through 40°.

Photographs of dye experiments for an angle of attack of 10°, in one-second intervals, were taken. In these plan-view images, the white circle is a circular underwater viewer, which was used for clear images below the water surface.

**Figure 9:**
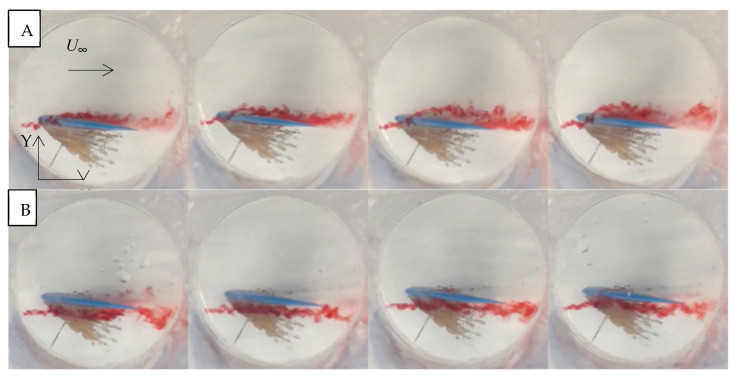
Plan view of the flow past the SFPW at α = 10° and *U*_∞_ = 0.5 mps using dye injections at the mid-section on the compression side (**A**) and the suction side (**B**). There was a 1 s time interval from left to right.

**Figure 10:**
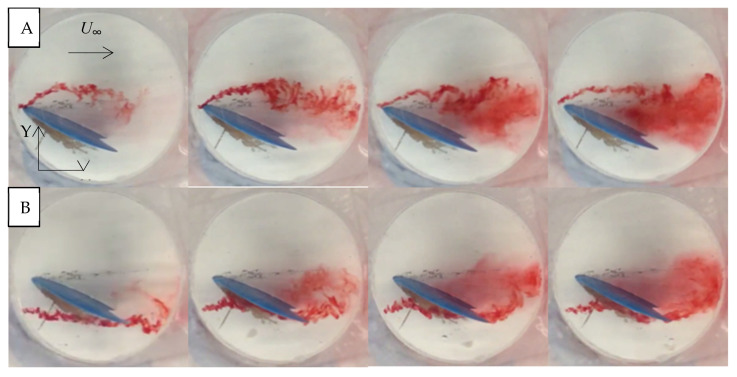
Plan view of the flow past the SFPW at α = 25°, *U*_∞_ = 0.5 mps using dye injections at the base section (**A**) compression side. Sequence from left to right (**B**) suction side. Sequence from left to right.

In the mid-section plan-view images, at 10° angles of attack, significant flow separation was not apparent on either side of the hydrofoil (Figure 9). The dye stayed attached to the fin as it moved over the surface. On the suction side, the dye avoided the leading edge and dispersed more as it moved towards the tail of the fin. For an angle of attack of 25°, there was significant flow separation and swirling eddies, as in the aforementioned CFD results (Figure 10).

**Figure 11:**
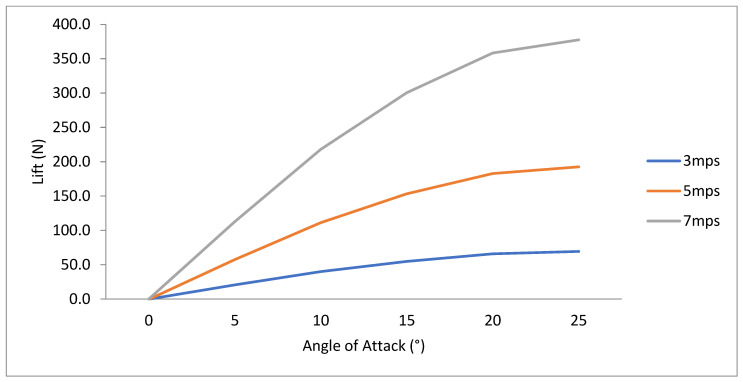
Lift (N) from CFD data collected for the SFPW at six angles of attack (0–25°) at three typical surfing velocities (*U*_∞_ = 3 mps, 5 mps, and 7 mps).

**Figure 12:**
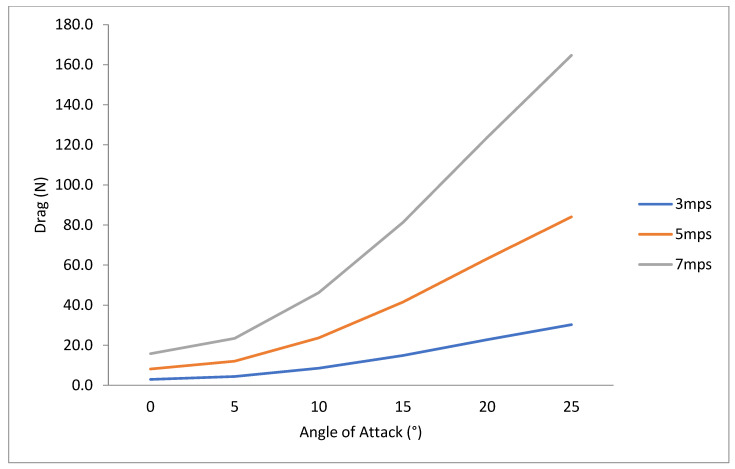
Drag (N) from CFD data collected for the SFPW at six angles of attack (0–25°) at three typical surfing velocities (*U*_∞_ = 3 mps, 5 mps, and 7 mps).

**Figure 13:**
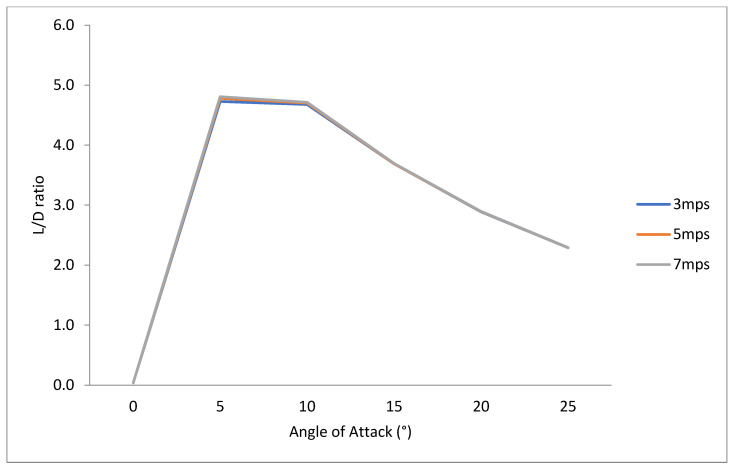
L/D ratio from CFD data collected for the SFPW at six angles of attack (0–25°) at three typical surfing velocities (*U*_∞_ = 3 mps, 5 mps, and 7 mps).

To investigate fin efficiency at higher-velocity values typical of prototype surfing conditions, numerical simulations were performed on typical surfing free-stream velocity values. As expected, higher lift and drag force values occurred at higher-velocity values (Figure 11 and Figure 12). The L/D ratio was insensitive to free-stream velocity, however (Figure 12). This insensitivity yields confidence that using the Short-Finned Pilot Whale fin shape at a 10° angle of attack is the most efficient for real-surfing velocity values (Figure 13).

Some additional considerations are as follows: (1) The fins were printed with 20% infill, which could affect the rigidity of the surfboard. However, the various fin shapes and angles of attack tested were attached to a small-scale surfboard based on that of a well-known board called the Gerry Lopez 1975 Lightning Bolt. This ensured the rigidity of a real surfboard. (2) PLS was chosen for the simulated fins, and modern surfboards use other materials. Future research should be performed with real surfboard materials, if possible, to determine how much of an effect this has on aerodynamic performance and overall characteristics. (3) Additional research is also warranted to determine if these results hold true for a full-size surfboard, especially given the potential differences in flow dynamics and Reynolds numbers. (4) Further research is needed to determine if these findings can be used for the highly unsteady flow conditions found in real surfing. This and other studies test under steady flow condition for simplicity.

## 4. Conclusions

Numerical simulations and physical laboratory experiments regarding flow around various bio-inspired surfboard fin shapes both show that the most efficient configuration of surfboard fin is a Short-Fin Pilot Whale fin at an angle of attack of 10°. Significant drag and flow-separation-induced eddies resulted in angles of attack greater than 25°. While there may be no new contribution to the field of hydrodynamics, since the fluid mechanics of flow around an airfoil closely resemble the flow around a surfboard fin, the contribution is, however, to the practical issue of choosing an angle of attack for the fin that maximizes the lift-to-drag ratio to allow for high surfboard speed and maneuverability.

These results are important because (1) this is one of the first studies on surfboard lift and drag forces both using CFD modeling and direct force measurement in a laboratory channel. (2) These results are bio-inspired, (3) they give an example of numerical and experimental techniques to successfully model flow around fins, and (4) they provide guidance on the highest lift-to-drag-ratio fin angle of attack for maximum lateral stability and fast forward speed. (5) Future research could include resolving the boundary layer instead of using wall functions, even though it is computationally expensive, since this has implications for wall shear stress.

## Data Availability

All data are available in [15].
